# Supplementary material for: Immediate unfavorable birth outcomes and determinants of operative vaginal delivery among mothers delivered in East Gojjam Zone Public Hospitals, North West Ethiopia: A cross-sectional study
Source: PLoS One. 2022 Jun 1;17(6):e0268782. doi: 10.1371/journal.pone.0268782 (PMC9159606; doi:10.1371/journal.pone.0268782)
Supplement: S1 File — (DOCX) [file pone.0268782.s001.docx]

**English questionnaire**

Hello! My name is _____________________I am one of the members of the research team, which has the objective of identifying determinants of immediate unfavorable birth outcomes of Operative Vaginal Delivery among Mothers Delivered in East Gojjam Zone Public Hospitals, North West Ethiopia.

As the study is directly related to outcomes of Operative Vaginal Delivery. And you may be one of the mothers who will be selected to participate in this study, therefore you are kindly requested to participate in this study and provide the information required from you. Your participation in this study is completely voluntary basis and you have the right to refuse from participating.

Your responses will be kept confidential and there will be no way of linking your responses to the final results of the study findings.

We would like to inform you that the responses that you provide to the questions are very essential, not only, for the successful accomplishment of this study, but also for producing relevant information which will help improve the immediate unfavorable birth outcomes of Operative Vaginal Delivery.

Are you willing to participate in this study to give your responses based on the questionnaire?

**Yes**____________signature________**No**___________

I understand the above information and I am happy to be a participant in the study by confirming with my signature.

Signature_______________

Name and Signature of the data collector _____________________

Name and signature of the supervisor ________________________

Date interview___________________________________________

Code Number __________________________________________

**Instruction:** for each of the following questions, please provide your appropriate response.

| **Part I ፡socio-demographic characteristic** | | |
| --- | --- | --- |
| **S.N** | **Question** | **Response** |
| 101. | Age | ………..years |
| 102. | Religion | 1. Orthodox 2. Islam 3. Protestant 4. Other----- |
| 103. | Marital status | 1. Single 2. Married 3. Divorced 4. Widowed |
| 104. | Educational level | 1. could not read and write  2. primary (1-8)  3. secondary(9-12)  4. college and above |
| 105. | Ethnicity | 1. Amhara  2. Oromo  3. Guragie  4. Tigre  5. Other…………… |
| 106. | Occupation | 1. Farmer 2. Merchant 3. Government employee 4. Private employee 5. Housewife 6. NGO employee 7. Student 8. Other______ |
| 107. | Residence | 1. Urban  2. Rural |
| 108. | Family income | ----------ETB |
| Part II: Obstetric Related Characteristics | | |
| 201. | How many times you got pregnant? | ____________(number) |
| 202. | How many of your pregnancy was viable (beyond 28 weeks of gestation)? | ____________(number) |
| 203. | What is the gestational age of this pregnancy? | ___________(weeks) |
| 204. | Do you have antenatal care follow-up? | 1. yes  2.no |
| 205. | If yes for question number 204, what was the gestational age when you start the visit? | --------------(weeks) |
| 206. | How many antenatal care visits do you have during the current pregnancy? | --------------(number) |
| 207. | Which danger sign/s happened during this pregnancy? (you can choose more than one option) | 1. No danger signs happened  2. vaginal bleeding before labor  3. Hypertension  4. Diabetes Mellitus  5. Cardiac Problem  6. Infection--------  7. Other ------------- |

| **Part III-Anthropometric Measurement** | | | |
| --- | --- | --- | --- |
| 301. | Weight of the mother | | ------------kg |
| 302. | MUAC | | ------------Cm |
| Part IV- Health professional related factors | | | |
| 401. | | Who assisted the delivery? | 1.Gynecologist  2.IEOS  3.Midwife  4.General practitioner  5.Health officer  6.Nurse  7.Other |
| 402. | | Experience of the Professional | -------month(year) |
| **Part V-Intra Partum Period** | | | |
| 501. | | Duration of second stage labor for primiparous | ----------min (hrs.’) |
| 502. | | Duration of the second stage for multiparous | ---------min(hrs) |
| 503. | | Cervical dilatation during the procedure | --------([40](#_ENREF_40)) |
| 504. | | Station at the time of instrument application | -------- |
| 505. | | Position at the time of instrument application | 1.Occipto Anterior  2.Occipto Lateral  3.Occipto Posterior |
| 506. | | What was the reason for instrumental delivery?  (more than one answer is possible) | 1. Poor maternal effort  2. Nonreassuring fetal heart rate pattern  3. Prolonged 2nd stage  4. Maternal medical condition  5. After coming head  6. Other |
| 507. | | Type of instrument used? | 1. Forceps  2. Vacuum  3. Both |
| 508. | | If vacuum was used, is it ------? | 1. Metalic  2. Plastic |
| 509. | | Type of instrument application? | 1.Outlet (Scalp Visible at introitus without separating labia )  2.Low( staton+2,+3)  3.Mid(station 0,+1) |
| 510. | | How many pulls were applied? | --------- |
| 511. | | Duration of the procedure (Instrumental delivery) | ------minute. |
| **Part VI- postpartum period- Maternal and neonatal condition** | | | |
| 601. | | Was episiotomy done? | 1. Yes  2.No |
| 602. | | Any laceration due to the procedure. What type of laceration/tear to the mother? (more than one answer is possible) | 1.No  2. Cervical tear  3. Vaginal tear------degree  4. Perineal tear.  A. First degree  B. Second degree  C. third degree  D. Fourth degree  5. Uterine rupture |
| 603. | | Does she have fecal or urinary incontinence after the procedure? | 1. Yes  2.No |
| 604. | | Postpartum hemorrhage due to the procedure? | 1. Yes  2.No |
| 605. | | Does she require a blood transfusion due to postpartum hemorrhage? | 1. Yes  2.NO |
| 606 | | Immediate maternal outcome | 1. Favorable  2. Unfavorable |
| 607. | | Apgar of the neonate in the 1st and 5th minute? | 1. -----2.-----respectively |
| 608. | | Sex of the neonate | 1. Male  2. Female |
| 609. | | Weight of neonate | --------gm. |
| 610. | | Does the neonate need resuscitation? | 1. Yes  2.No |
| 611. | | Does the neonate refer to neonatal ICU? | 1. Yes  2.No |
| 612. | | If question number 611 is yes, what was the indication for the referral? | ---------- |
| 613. | | Is there any procedural trauma to the neonate? (more than one answer is possible) | 1.NO  2. Scalp laceration  3. skull fracture  4. Facial nerve palsy  5. subgalial hemorrhage  6. cephalhematoma  7. Retinal hemorrhage  8. Others--------- |
| 614. | | Immediate fetal outcome | 1. Favorable  2. Unfavorable |

***Thank You for your time!***
