# Supplementary material for: Immediate unfavorable birth outcomes and determinants of operative vaginal delivery among mothers delivered in East Gojjam Zone Public Hospitals, North West Ethiopia: A cross-sectional study
Source: PLoS One. 2022 Jun 1;17(6):e0268782. doi: 10.1371/journal.pone.0268782 (PMC9159606; doi:10.1371/journal.pone.0268782)
Supplement: Attachment — Submitted filename: RE-PONE-D-21-32649.docx [file pone.0268782.s002.docx]

**RE: PONE-D-21-32649**
Unfavorable Feto-Maternal Outcomes and Associated Factors of Operative Vaginal Delivery among Women Delivered in East Gojjam Zone Hospitals, North West Ethiopia
PLOS ONE

**COMMENTS TO THE EDITOR:**

I think it is an interesting study, within the scope of the journal, that addresses a clinically relevant aspect: factors associated to adverse feto-maternal outcomes in operative vaginal deliveries.

Furthermore, I believe it is a topic of special interest in developing countries such as Ethiopia, where health resources can be scarce, and therefore identifying correctly possible risk factors for adverse neonatal and maternal outcomes is crucial in order to reduce morbidity and mortality.

However, the English language used is confusing and makes the manuscript difficult to follow and understand. I consider that the article requires a thorough linguistic revision so that it can be made understandable in order to assess other aspects of the study.

**COMMENTS TO THE AUTHORS:**

In first place, I would like to congratulate the authors for conducting a study evaluating the relationship between adverse maternal outcomes and adverse neonatal outcomes in operative vaginal deliveries, as it is a clinically relevant topic that requires research in order to improve health assistance.

The title ‘Unfavorable Feto-Maternal Outcomes and Associated Factors of Operative Vaginal Delivery among Women Delivered in East Gojjam Zone Hospitals, North West Ethiopia’ describes correctly the purpose of the study.

I believe the study has several highlights:

- It is a multicenter study carried out in nine different hospitals, reaching a consecutive sample of pregnant women who delivered by either forceps or vacuum in a short perio of time (March 1 to April 30, 2019).

- Statistical design including sample size calculation and sampling procedure is well described in the ‘Methods’.

- Analysis of possible risk factors associated with adverse maternal and neonatal outcomes is thoroughly described.

- Tables 3 and 4 are nicely presented and easy to understand.

However there are some issues I believe the authors must address.

MAJOR ISSUES:

- the English language used is confusing and makes the manuscript difficult to follow and understand. I suggest a thorough linguistic revision by native English speaker so that the paper is more approachable and other aspects of the study can be addressed.

- The title, although describes the main idea of the study is long and confusing. I recommend reviewing.

-The abstract does not include the main objective of the study in a separate subsection. Within the ‘Background’, the authors state: ‘Furthermore, no study reported both maternal and neonatal outcomes at a time.’ Are the authors certain of this statement?

- Introduction:

Authors state (lines 56-58): ‘Neonatal trauma is associated with initial unsuccessful attempts at operative vaginal delivery by inexperienced operators.’ Are the authors certain of this statement? Could they provide reference?

- Objective of the study is not clearly described.

- Methods:

This section is divided into multiple subsections (Study area, design and period; Study design; Source population; Inclusion and exclusion criteria; Sample size and sampling procedure; Sampling procedure; Study variables; Operational definitions; Data quality-control; Daa processing and Analysis and Ethical clearance). These sections make the ‘Methods’ of the study unclear and confusing. Furthermore, similar information is repetead in the different subsections, for example ‘Source population’ and ‘Study population’

Sampling procedure: Authors state that mothers and neonates were followed for treatment and obstetrical care outcomes until discharged (six hours) from each selected hospital. I believe six hours is a very short period of time to report accurately the rate of adverse maternal and neonatal outcomes.

Are subsequent Emergency visits to the hospital after discharge evaluated in these patients?

- Results:

I suggest changing ‘Result’ for ‘Results’.

In this particular section, English is really confusing, making it really difficult to follow the results the authors obtain (example: Line 215 ‘Greater than three fourth (77%) of the respondents were…’).

Terms such as ‘asphyxia’ should be well defined before being included in the results section.

Some phrases are repeated practically identically in different paragraphs (example: Line 242: Finally, educational status, residence, sex of the neonate …..’. Line 246 Educational level […], resident […]’)

- Discussion:

Again, English used throughout the section is confusing, being difficult to understand what the authors are trying to discuss.

References are missing in order to provide evidence for certain statements such as (Line 289): The reason might be male fetus has the probability of being post-term pregnancy and as a result weight might be increased than female fetus which increase the bad maternal outcome of operative vaginal delivery.’

- Limitation:

Only one limitation is stated in this section. I believe the study has several more limitations that the authors should state in this section.

- Conclusion:

The first sentence of the ‘Conclusion’ is confusing: This study revealed that the overall unfavorable maternal and neonatal outcomes of operative delivery are found to be higher in East Gojjam Zone hospitals.’ Higher than what?

- References do not always respect Vancouver style.

- Tables:

Table 2 is too long and difficult to follow.
