# Supplementary material for: Immediate unfavorable birth outcomes and determinants of operative vaginal delivery among mothers delivered in East Gojjam Zone Public Hospitals, North West Ethiopia: A cross-sectional study
Source: PLoS One. 2022 Jun 1;17(6):e0268782. doi: 10.1371/journal.pone.0268782 (PMC9159606; doi:10.1371/journal.pone.0268782)
Supplement: Attachment — Submitted filename: Response to reviewer1 H final.docx [file pone.0268782.s003.docx]

**Response to reviewers**

**Dear reviewers, I would like to forward our gratitude to you for such constructive comments and suggestions you gave. We have managed the manuscript based on the comments and suggestions you gave and here below are responses to your questions.**

1. **Reviewer #1**

- Based on your recommendation, we have tried to see the language with grammarly and managed accordingly.
- The title is also revised and corrected.
- Abstract was revised based on your recommendation and highlighted on “track changed manuscript” for correction and corrected on the clean manuscript.
- **‘Furthermore, no study reported both maternal and neonatal outcomes at a time.’ Are the authors certain of this statement?**
  - Yes, we have searched a lot but we could not find a similar study in the study area.
- **Introduction** is corrected based on the given comments
- **Methods**
  - Corrected based on your recommendation
- **Are subsequent Emergency visits to the hospital after discharge evaluated in these patients?**
- We didn't visit after discharge because our objective was to assess immediate maternal and neonatal outcomes.
- **Results**
  - We have corrected based on the comments and suggestions given
- **Terms such as ‘asphyxia’ should be well defined before being included in the results section**
  - We operationalized terms that had a different meaning in the study. Asphyxia is a scientifically known term that had a no different meaning in the study.
- **Discussion**
  - Corrected
- **Limitation**
- **Only one limitation is stated in this section. I believe the study has several more limitations that the authors should state in this section.**
  - Based on our view we could not get any limitation other than the limitation we wrote.
- **Conclusion**
  - **Corrected**
- **Table 2 is too long and difficult to follow.**
  - All variables deal with one characteristic (obstetric) so that we cannot split it into two tables.
- **Reference style** is Vancouver(we have used automatic endnote)

2. **Reviewer #2**

- **Abstract**
- Line 10: corrected
- Line 14: corrected
- Line 21: corrected
- Results: AOR was fully interpreted in discussion part
- **Introduction**
- Line 39: corrected
- Line 40: corrected
- Line 43: corrected
- Line 47: corrected
- Line 56: corrected
- **Methods**
- Data collectors are described in the manuscript
- We excluded the confounding factors like congenital anomalies from the study
- **Result and discussion**
- Grammatical errors are corrected based on your suggestions
